# The Cassava NBS-LRR Genes Confer Resistance to Cassava Bacterial Blight

**DOI:** 10.3389/fpls.2022.790140

**Published:** 2022-02-01

**Authors:** He Zhang, Zi Ye, Zhixin Liu, Yu Sun, Xinyu Li, Jiao Wu, Guangzhen Zhou, Yinglang Wan

**Affiliations:** ^1^Key Laboratory of Integrated Pest Management on Tropical Crops, Ministry of Agriculture and Rural Affairs, Environment and Plant Protection Institute, Chinese Academy of Tropical Agricultural Sciences, Haikou, China; ^2^Hainan Key Laboratory for Sustainable Utilization of Tropical Bioresources, College of Tropical Crops, Hainan University, Haikou, China

**Keywords:** cassava, cassava bacterial blight, resistance genes, salicylic acid, ROS, NBS-LRR

## Abstract

Cassava bacterial blight (CBB) caused by *Xanthomonas axonopodis* pv. *manihotis* (*Xam*) seriously affects cassava yield. Genes encoding nucleotide-binding site (NBS) and leucine-rich repeat (LRR) domains are among the most important disease resistance genes in plants that are specifically involved in the response to diverse pathogens. However, the *in vivo* roles of *NBS-LRR* remain unclear in cassava (*Manihot esculenta*). In this study, we isolated four *MeLRR* genes and assessed their expression under salicylic acid (SA) treatment and *Xam* inoculation. Four *MeLRR* genes positively regulate cassava disease general resistance against *Xam* via virus-induced gene silencing (VIGS) and transient overexpression. During cassava-*Xam* interaction, *MeLRRs* positively regulated endogenous SA and reactive oxygen species (ROS) accumulation and pathogenesis-related gene 1 (*PR1*) transcripts. Additionally, we revealed that *MeLRRs* positively regulated disease resistance in *Arabidopsis*. These pathogenic microorganisms include *Pseudomonas syringae* pv. *tomato*, *Alternaria brassicicola*, and *Botrytis cinerea*. Our findings shed light on the molecular mechanism underlying the regulation of cassava resistance against *Xam* inoculation.

## Introduction

Disease resistance genes (*R* genes) usually act as receptors of pathogen-encoded effector proteins, which are often secreted by pathogens directly into host cells (Urbach and Ausubel, 2017). *R* genes are specifically involved in the response to diverse pathogens, including fungi, bacteria, viruses, nematodes, insects, and oomycetes (Dalio et al., 2017). In the past 30 years, more than 300 *R* genes have been cloned from many plant species (Kourelis and van der Hoorn, 2018). Among them, genes encoding nucleotide-binding site (NBS) and leucine-rich repeat (LRR) domains are important *R* genes in plants (van der Hoorn and Kamoun, 2008; Pandolfi et al., 2017). The amino terminal (*N*-terminal) of NBS-LRR proteins usually contain the Toll/interleukin-1 receptor-like (TIR) domain, coiled-coil (CC) domain, or resistance to powdery mildew 8 (RPW8) domain, and the carboxyl terminus (C-terminus) contain a zinc-finger transcription factor-related domain containing the WRKY sequence (WRKY domain) (Shao et al., 2006). Based on the *N*-terminal domains, *NBS-LRR* was usually divided into three subclasses, namely TIR-NBS-LRR (TNL), CC-NBS-LRR (CNL), and RPW8-NBS-LRR (RNL) proteins (Shao et al., 2006).

In plant genome, about 0.2–1.6% of genes are predicted as *NBS-LRR-*coding genes (Jia et al., 2015). For instance, there are 150–175 *NBS-LRR* genes in *Arabidopsis thaliana* genome (Meyers et al., 2003; Joshi et al., 2011), constituting about 0.6% of its 25,000 genes, and there are approximately 600 *NBS-LRR* genes in rice (*Oryza sativa* ssp. *japonica*) genome (Goff et al., 2002; Chen et al., 2015), constituting about 1.5% of its 40,000 genes (Goff et al., 2002). In the past few years, *NBS-LRR* genes in several plant species have been isolated via genome-wide analysis, including mango (*Mangifera indica*) (Lei et al., 2014), cassava (*Manihot esculenta*) (Lozano et al., 2015; Utsumi et al., 2016), sorghum (*Sorghum bicolor*) (Yang and Wang, 2016), wheat (*Triticum aestivum*) (Li et al., 2017), cotton (*Gossypium hirsutum*) (Deng et al., 2019), maize (*Zea mays*) (Xu et al., 2018), soybean (*Glycine max*) (Zhao et al., 2018), grapevine (*Vitis vinifera*) (Goyal et al., 2020), and yam (*Dioscorea rotundata*) (Zhang et al., 2020). In recent years, accumulated evidence has confirmed that NBS-LRR protein is widely involved in plant development and stress response (Li et al., 2017, 2018a,2018b; Xu et al., 2018; Zhao et al., 2018; Deng et al., 2019). Activated by effector proteins, NBS-LRR proteins could elicit robust defense responses, inducing the biosynthesis and accumulation of SA and increasing expression of pathogenesis-related (*PR*) genes (Wu et al., 2014; Palmer et al., 2019). In cotton, silencing of the *NB-ARC* domain-containing (*GbaNA1*) gene impaired cotton resistance to *Verticillium dahliae* Vd991 (Li et al., 2018a). Similarly, heterologous expression of the maize *NBS-LRR* gene *ZmNBS25* enhanced resistance to *P. syringae* pv. *tomato* DC3000 in rice and *Arabidopsis* by induced the defense-related gene expression, but grain yield was not affected (Xu et al., 2018). NBS-LRR proteins and SA are involved in pathogen-host interactions (Bonardi et al., 2011; Zhao et al., 2018). Yoodee et al. (2018) found that exogenous application of SA could elevate the defense resistance of cassava to *Xam* inoculation. Although 228 *NBS-LRR* genes have been identified in cassava (Lozano et al., 2015), their role remains unknown *in vivo*.

Cassava is a widely grown drought-tolerant crop that can be cultivated as an annual crop in marginal soils in tropical and subtropical regions of the world (Lozano et al., 2015; Bredeson et al., 2016). However, as a clonally propagated crop, cassava is especially vulnerable to pathogens, especially cassava bacterial blight (*X. axonopodis* pv. *manihotis* = *X. phaseoli* pv. *manihotis*) (Bredeson et al., 2016; Constantin et al., 2016; Zárate-Chaves et al., 2021), cassava brown streak disease (*Cassava brown streak virus*, CBSV) and anthracnose disease (*Colletotrichum gloeosporioides*) (Utsumi et al., 2016). Therefore, it is best to identify the NBS-LRR proteins in cassava. Results presented by Utsumi et al. (2016) indicated that the transcript level of *NBS-LRRs* was inducted by *C. gloeosporioides* infection. Similar results were obtained under plants infected by viruses (Louis and Rey, 2015; Lozano et al., 2015; Amuge et al., 2017; Masumba et al., 2017). A cluster of *NBS-LRR* genes on chromosome 11 of cassava genome was associated with resistance to cassava brown streak disease via genome-wide associated mapping and genomic selection (Kayondo et al., 2018). However, the mechanisms remain unclear, particularly in experimental investigation and verification.

In this study, we analyzed the published transcriptome databases of cassava-pathogens interaction (Lozano et al., 2015; Utsumi et al., 2016). Within the database, four *NBS-LRR* genes that showed high transcription level after pathogen infection attracted our attention. The expression levels of four chosen *MeLRRs* were significantly induced by exogenous application of SA treatment and *Xam* inoculation. Moreover, these genes positively regulated cassava resistance to *Xam* inoculation. The functional analysis of *MeLRR* genes will offer potential roles in genetic breeding for disease-resistant cassava.

## Results

### Identification of the Cassava Bacterial Blight Resistance Locus in Cassava

There are 228 *NBS-LRRs* in cassava, including both *TIR-NBS-LRR* and *CC-NBS-LRR*. Their transcript levels were analyzed through RNA-seq in response to CBSV and *C. gloeosporioides* infection (Lozano et al., 2015; Utsumi et al., 2016). Among these, four *MeLRRs* (*MeLRR1*, *MeLRR2*, *MeLRR3*, and *MeLRR4*) were both induced under CBSV and *C. gloeosporioides* infection and selected for further analysis. The four MeLRR proteins have typical leucine-rich repeats, which are named MeLRR1 (Manes. 11G053000.1), MeLRR2 (Manes. 03G071700.1), MeLRR3 (Manes. 13G036800.1), and MeLRR4 (Manes. 07G107800.1), located on chromosomes 11, 3, 13, and 7, respectively. MeLRR1, MeLRR3, and MeLRR4 belong to CC-NBS-LRR protein, while MeLRR2 is one of the TIR-NBS-LRR protein. Bioinformatics predicted that the MeLRR proteins were unstable and hydrophilic (Supplementary Table 1). The phylogenetic analysis showed that MeLRR1 clustered with XP_012073222.1 of *Jatropha curcas*, MeLRR2 clustered with XP_021684995.1 of *Hevea brasiliensis*, MeLRR3 clustered with XP_020535356.1 of *J. curcas*, and MeLRR4 clustered with KAF2295929.1 of *H. brasiliensis* based on whole protein sequences (Supplementary Figure 1).

### Subcellular Localization of the *MeLRR* Proteins

**FIGURE 1:**
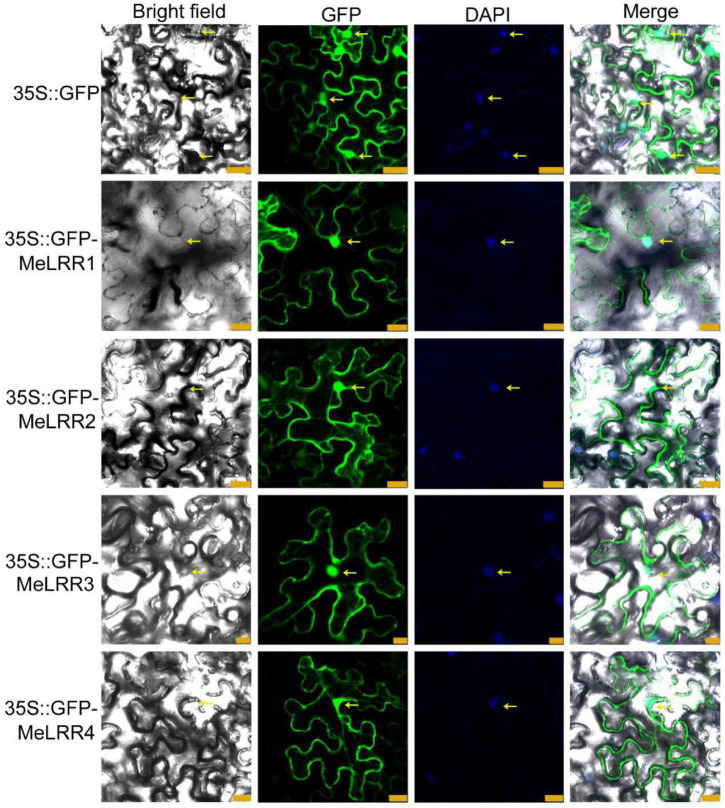
Subcellular localization of MeLRR proteins in *N. benthamiana* leaves. Transient expression of Agrobacterium GV3101 with *35S:GFP* and *35S:GFP-MeLRRs* plasmids in *N. benthamiana* leaves. After 2 dpi, the fluorescence was scanned by a Leica confocal microscopy system (Leica TCS SP8, Solms, Germany) with an excitation wavelength of 488 nm and a 505–530 nm bandpass emission filter. The empty vector *35S:GFP* was used as a control. Nuclei were stained using DAPI (4’,6-diamidino-2-phenylindole). Scale bar = 25 μm.

To investigate the subcellular localization of the MeLRR proteins, the coding sequences (CDSs) of MeLRRs were cloned and inserted into the poly-cloning sites of the fusion expression vector pEGAD and fused upstream to a green florescence protein (GFP) fusion partner by the constitutive *CaMV35S* promoter. The *Agrobacterium tumefaciens* strain GV3101 cell culture harboring the pEGAD empty vector containing *35S:GFP* was used as a control, and tobacco (*Nicotiana benthamiana*) leaves were infected with *35S:GFP* or *35S:GFP-MeLRR1,−2,−3,−4* plasmid as described by Sparkes et al. (2006). The fluorescence of transiently expressing MeLRR proteins in tobacco leaf epidermal cells was detected in the nucleus, cytoplasm and cytomembrane, similar to that of *35S:GFP* (Figure 1).

### Expression Level of *MeLRR* Genes in Response to SA Treatment and *Xam* Inoculation

**FIGURE 2:**
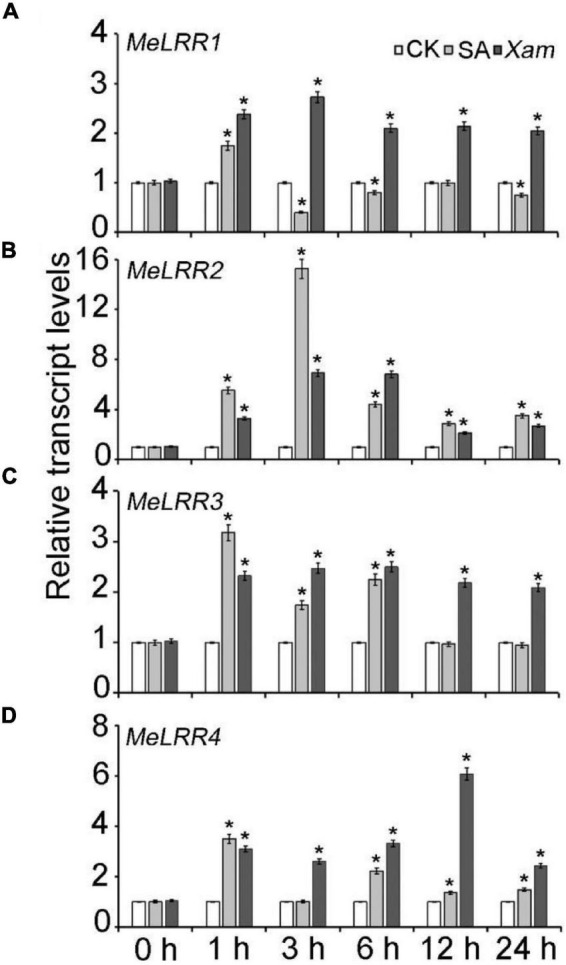
The expression profiles of *MeLRR* genes in cassava in response to SA treatment and *Xam* inoculation. For the assays, 4-week-old cutting seedlings of cassava leaves were sprayed with water, 5 mmol/L salicylic acid, or 4 × 10^8^ cfu/mL *Xam* suspension for 0, 1, 3, 6, 12, or 24 h. The transcript levels of *MeLRR1* **(A)**, *MeLRR2* **(B)**, *MeLRR3* **(C)**, and *MeLRR4* **(D)** at 0 h of the mock treatment was normalized to 1. Asterisks (*) indicate significant differences at *p* < 0.05.

The expression profile of *MeLRRs* in response to SA treatment and *Xam* inoculation were analyzed by qRT-PCR (real-time quantitative reverse transcription PCR). Under SA treatment, the expressions of *MeLRR1*, *MeLRR3*, and *MeLRR4* were induced and peaked at 1 h post treatment (hpt), while *MeLRR2* showed the highest level at 3 hpt (Figure 2). Following infection by *Xam*, the expression level of *MeLRRs* at 1–24 hpt hpi was significantly higher than that at 0 hpi (Figure 2). Moreover, the expression of *MeLRR1*, *MeLRR2*, and *MeLRR3* were induced and peaked at 3 hpt, while the expression of *MeLRR4* reached the peak at 12 hpt (Figure 2).

### Virus-Induced Gene Silencing of *MeLRR* Genes

**FIGURE 3:**
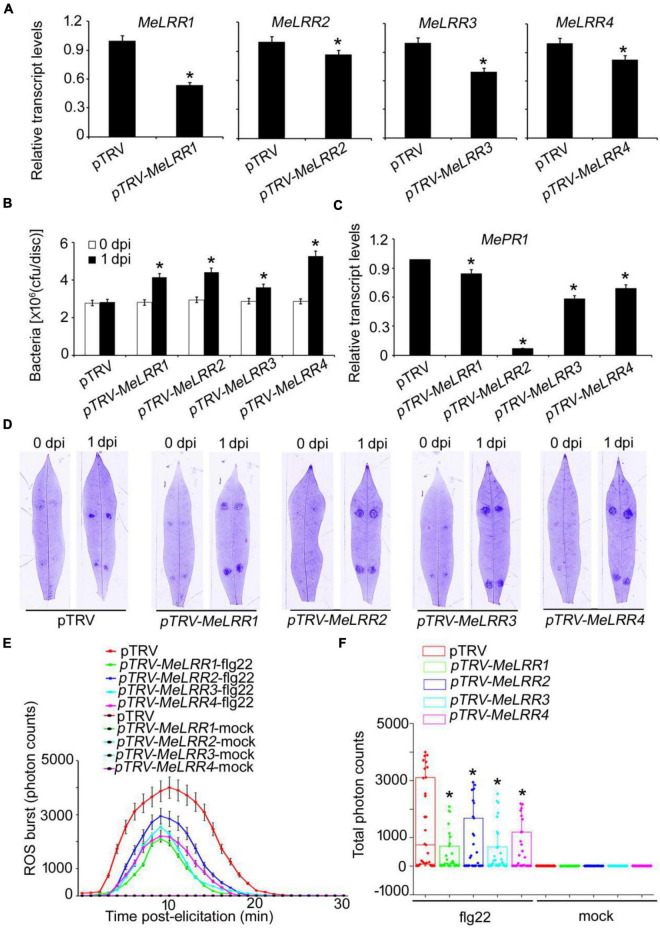
The VIGS of *MeLRRs* reduced disease resistance against cassava bacterial blight. **(A)** At 14 dpi, the new leaves were used for relative transcript levels of *MeLRRs* in *MeLRR*-silenced leaves and the pTRV control leaves. Then, the new leaves were syringe infiltrated with 4 × 10^8^ cfu/mL of pathogenic bacteria *Xam* used for disease resistance assay. **(B)** The number of *Xam* populations in *MeLRR-*silenced cassava and the pTRV control leaves at 0 and 1 dpi, respectively. **(C)** The pathogenesis-related gene (*MePR1*) transcript level was quantitatively analyzed by qRT-PCR at 1 dpi. The relative transcript level of *MePR1* in the pTRV control leaves was normalized to 1.0. **(D)** Cassava leaves were observed using a Coomassie brilliant blue imaging system Fusion FX7-826 apparatus (Vilber Lourmat, France). **(E)** Dynamic of ROS accumulation in response to flg22 elicitation in *MeLRR-*silenced cassava and the pTRV control leaves. The flg22-triggered ROS burst were measured using luminol-based assay by a GloMax 96 Microplate Luminometer. **(F)** Total photon of *MeLRR-*silenced cassava and the pTRV control leaves. Multiple comparisons of total photon were calculated by Student’s *t*-test. Asterisks (*) indicate significant differences at *p* < 0.05. dpi is days post-infection.

To analyze the function of *MeLRRs*, we constructed *MeLRR*-silenced cassava plants by virus-induced gene silencing (VIGS). The partial sequences of *MeLRR1* (453 bp), *MeLRR2* (441 bp), *MeLRR3* (433 bp) and *MeLRR4* (423 bp) were individually inserted into pTRV2 plasmid to construct VIGS vector. At 14 days post-infection (dpi) in cassava infected with Agrobacterium GV3101 carrying the pTRV*-MeLRR* plasmids, qRT-PCR was performed to detect the target gene transcript level. The transcript level of the target *MeLRR-1*,*-2*,*-3*,*-4* genes were significantly decreased in the *MeLRR-*silenced cassava leaves compared to the pTRV empty vector. The silencing efficiency of *MeLRR-1*,*-2*,*-3*,*-4* was 46.33 (± 2.31)%, 15.28 (± 0.49)%, 30.22 (± 2.28)%, and 17.45 (± 0.87)% (Mean ± SD, *n* = 3), respectively (Figure 3A). It was noteworthy that the silenced of *MeLRR1* did not affected the transcription of *MeLRR2*,*-3*,*-4*. Similar results were verified in *MeLRR2*-, *MeLRR3*-, and *MeLRR4-*silenced plants (Supplementary Figure 2). When co-silenced four target genes (*MeLRR-1*,*-2*,*-3*,*-4*) in one VIGS line, the transcript levels of all four target genes were significantly decreased (Supplementary Figure 3). On the contrary, the bacteria number was significantly higher than that in the pTRV empty vector-infected cassava leaves (Figure 3B). Moreover, the cassava *MePR1* transcript level was significantly decreased (Figure 3C). And the transcript level of *MePR1* in pTRV-*MeLRR1,−2,−3,−4* cassava was reduced on average to 84, 7, 58, and 69%, respectively, of the transcript level in the pTRV control at 14 dpi (Figure 3C). Additionally, the transcript level of *MePR1* in *MeLRR-1*,*-2*,*-3*,*-4-*silenced plant was significantly reduced to 66% (Supplementary Figure 3). Silencing of *MeLRRs* conferred increased disease susceptibility in cassava leaves (Figure 3D). Moreover, *MeLRRs-*silenced cassava leaves showed significantly lower ROS burst measurements than the empty vector (Figures 3E,F). These results indicate that silencing of *MeLRRs* impairs cassava resistance to *Xam*.

### Transient Overexpression of *MeLRR* Genes

**FIGURE 4:**
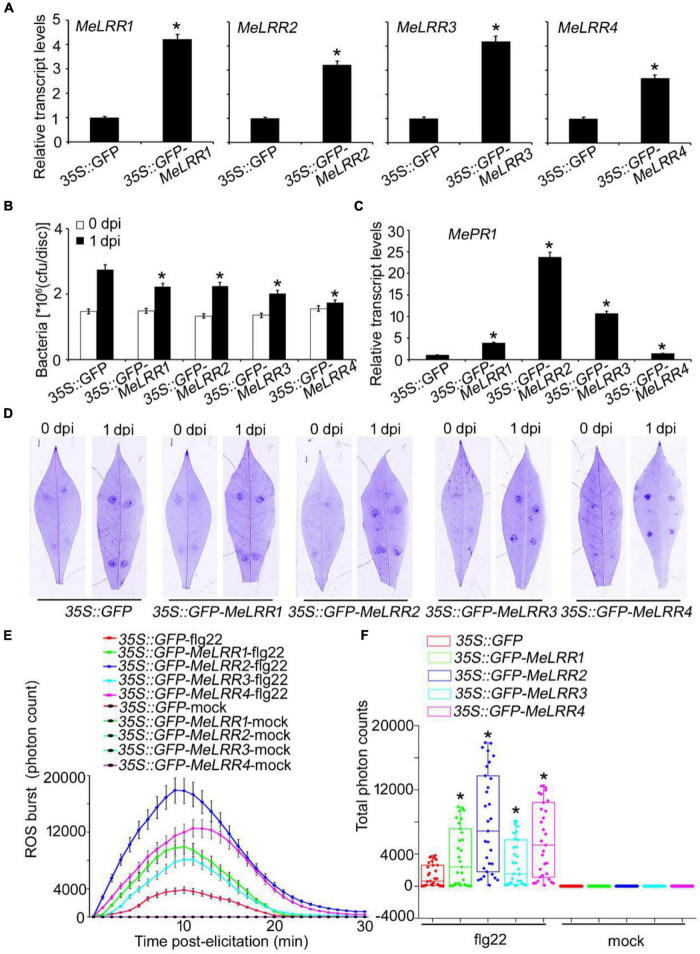
Transient overexpression of *MeLRRs* improved disease resistance against cassava bacterial blight. Cassava leaves inject with recombinant pEGAD plasmids and empty vector of Agrobacterium GV3101, respectively. **(A)** At 3 days later, the relative transcript levels of *MeLRRs* in *MeLRR-*overexpression cassava and the pEGAD control leaves. The relative transcript levels of *MeLRRs* in the pEGAD control leaves was normalized to 1.0. Then, the cassava leaves were syringe infiltrated with 4 × 10^8^ cfu/mL of pathogenic bacteria *Xam* used for disease resistance assay. **(B)** The number of *Xam* populations in *MeLRR-*overexpression cassava and the pEGAD control leaves at 0 and 1 dpi, respectively. **(C)** The pathogenesis-related gene (*MePR1*) transcript level was quantitatively analyzed by qRT-PCR at 1 dpi. The relative transcript level of *MePR1* in the pEGAD control leaves was normalized to 1.0. **(D)** Cassava leaves were observed using a Coomassie brilliant blue imaging system Fusion FX7-826 apparatus (Vilber Lourmat, France). **(E)** Dynamics of ROS accumulation in response to flg22 elicitation in *MeLRR-*overexpression cassava and the pEGAD control leaves. The flg22-triggered ROS burst were measured using luminol-based assay using a GloMax 96 Microplate Luminometer. **(F)** Total photon of *MeLRR-*overexpression cassava and the pEGAD control leaves. Multiple comparisons of total photon were calculated using Student’s *t*-test. Asterisks (*) indicate significant differences at *p* < 0.05. dpi is days post-infection.

To further verify the function of *MeLRRs*, *35S:GFP-MeLRR* recombinant plasmids were constructed and introduced into Agrobacterium strain GV3101. Cassava leaves were infected with *Agrobacterium* containing the recombinant plasmids or empty vector for 3 days. The transcript level of the target *MeLRR-1*,*-2*,*-3*,*-4* genes were significantly higher than that in the *35S:GFP* empty vector (Figure 4A). It is similar in silenced plant, overexpressing *MeLRR1* plant did not affected the transcription of *MeLRR2*,*-3*,*-4*, and the same as in *MeLRR2*-, *MeLRR3*-, and *MeLRR4*-overexpressed plants (Supplementary Figure 4). However, the transcript levels of the four target genes were significantly enhanced in co-overexpression *MeLRR-1*,*-2*,*-3*,*-4* plants (Supplementary Figure 5). On the contrary, the bacteria number was significantly lower than that in the control (Figure 4B). However, the transcript levels of *MePR1* in *35S:GFP-MeLRR1,−2,−3,−4* cassava were increased by 3. 77-, 23. 73-, 10. 70-, and 1.39-fold, respectively, compared to those in the control at 3 dpi (Figure 4C). Similarly, the transcript level of *MePR1* in co-overexpression *MeLRR-1,−2,−3,−4* lines was significantly increased by 24.03-fold (Supplementary Figure 5). Interestingly, overexpression of *MeLRRs* conferred improved disease resistance in cassava leaves (Figure 4D). Moreover, cassava leaves that overexpressed *MeLRRs* exhibited significantly higher ROS burst than *35S:GFP* control during flg22 treatment (Figures 4E,F). These results suggest that *MeLRRs* positively regulated cassava resistance to *Xam*. In addition, trypan blue staining showed no cell death phenotype at 2 dpi at transient expression of *MeLRRs* in cassava and *N. benthamiana* leaves (Supplementary Figure 6).

### *MeLRR*-Mediated Cassava Immune Responses via SA Accumulation

**FIGURE 5:**
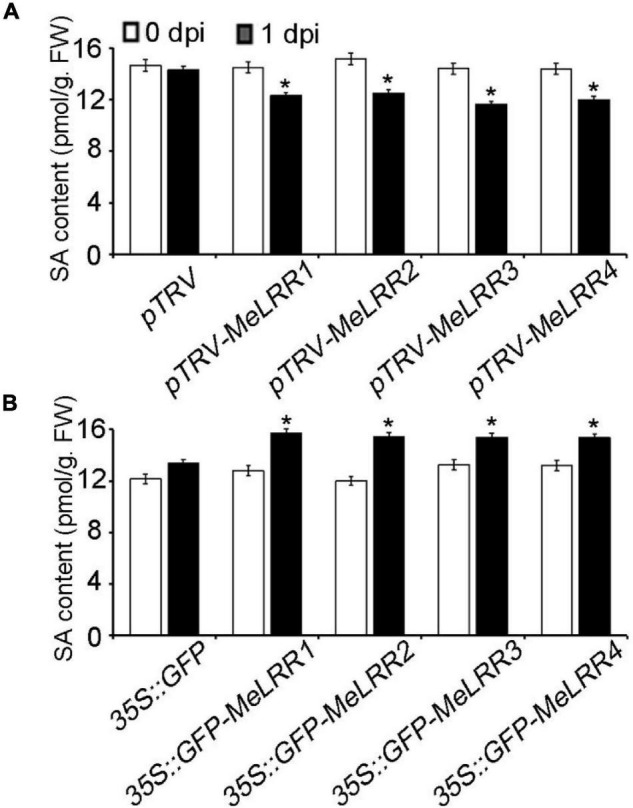
The salicylic acid content in cassava. The SA content in *MeLRR*-silenced **(A)** and *MeLRR*-overexpression **(B)** cassava leaves. Transient expression of *MeLRRs* conferred immunity. At 0 and 1 d infection with *Xam*, the cassava leaves were used for analyzing the content of SA. Asterisks (*) indicate significant differences at *p* < 0.05. dpi is days post-infection.

To further analyze the mechanism of *MeLRRs* in response to *Xam* inoculation, the SA content was measured. As shown in Figure 5, the SA level in *MeLRR1,−2,−3,−4*-silencing was significantly decreased compared with that in pTRV control cassava leaves (Figure 5A). By contrast, the SA level in *MeLRRs* overexpression was significantly increased compared with the control cassava leaves (Figure 5B). These results suggested that *MeLRR1,−2,−3,−4* positively participated in cassava immune responses via SA accumulation.

### Overexpression of *MeLRR* Genes in *Arabidopsis* Enhances Resistance to Plant Pathogens

**FIGURE 6:**
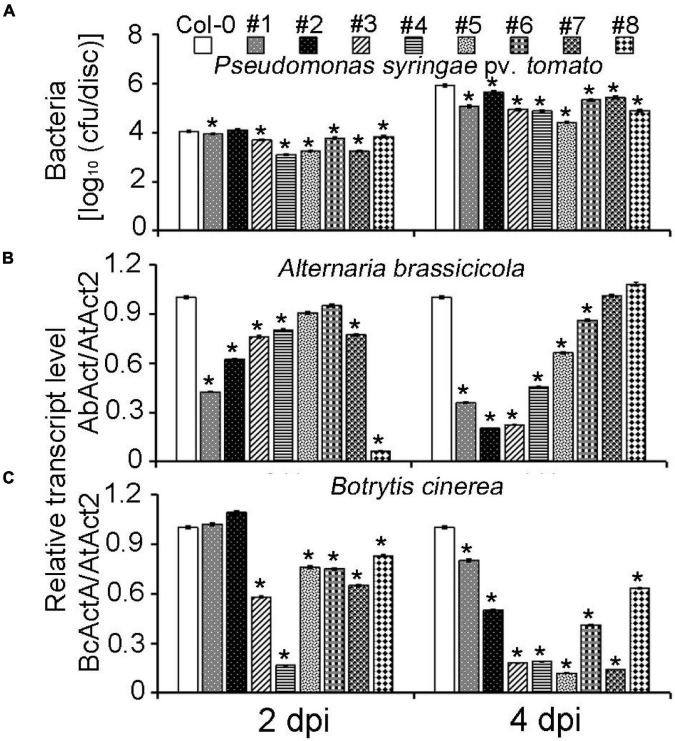
Overexpression of *MeLRRs* in *Arabidopsis* enhances resistance to plant pathogens. **(A)** The number of *P. syringae* pv. *tomato* populations in overexpression *Arabidopsis* leaves and the wild type. The relative transcript levels of *AbAct/AtAct2* **(B)** and *BcActA/AtAct2* **(C)** in overexpression *Arabidopsis* leaves and the wild type after infection with *A. brassicicola* and *B. cinerea*, respectively. Asterisks (*) indicate significant differences at *p* < 0.05. Col-0 is *A. thaliana* ecotype Columbia-0. #1 and #2, #3 and #4, #5 and #6, and #7 and #8 are overexpression of MeLRR3 in *A. thaliana* Col-0 lines, respectively. dpi is days post-infection.

To further confirm the *MeLRR* function, *MeLRRs* were overexpressed in *Arabidopsis*. Quantification of endogenous SA levels indicated that *MeLRR-*overexpressing lines accumulated significantly higher levels than WT leaves (Supplementary Figure 7). The *MeLRRs* overexpression plants displayed slight symptoms of wilting in response to *P. syringae* pv. *tomato*, *A. brassicicola*, and *B. cinerea* infection support the hypothesis that *MeLRRs* functions in a pathogen response pathway. A difference was already observed in the WT, suggesting that restricted bacterial entry into the leaves may underlie part of the apparent resistance (Figure 6A). Unlike *P. syringae* pv. *tomato*, *A. brassicicola*, and *B. cinerea* can enter hosts by penetrating the cuticle. Consistently, there was less fungal growth in leaves overexpressing these factors than WT plants by analyzing the transcript levels of the *A. brassicicola AbAct* (JQ671669.1) gene and *B. cinerea BcActA* (XM_024697950.1) gene (Liao et al., 2016) with the *Arabidopsis AtAct2* gene as an internal control at 2 and 4 dpi, respectively (Figures 6B,C).

To determine whether the enhanced resistance to plant pathogens was related to changing the defense response genes expression level, we used qRT-PCR to analyze the expression levels of *AtICS1*, *AtPDF1.2*, *AtPR1*, *AtPR2*, *AtPR5*, and *AtTGA3* in WT and *MeLRR* overexpression lines upon *A. brassicicola*, *B. cinerea*, and *P. syringae* pv. *tomato* DC3000 infection (Supplementary Figure 8). Particularly, the relative expression levels of genes involved in the SA synthesis pathway and pathogen resistance showed higher level in overexpression *MeLRR1* and *MeLRR2* in *Arabidopsis* plants than in control plants without *A. brassicicola*, *B. cinerea*, and *P. syringae* pv. *tomato* DC3000 infection. Similar results were observed in plant pathogen-infected overexpression of *MeLRR3* in *Arabidopsis* plants compared with control plants. However, *AtPDF1.2* and *AtPR1* were significantly down-regulated in overexpression of *MeLRR3* in *Arabidopsis* plants than in control plants without *P. syringae* pv. *tomato* DC3000 infection. On the other hand, the expression levels of *AtPR2* and *AtTGA3* were significantly up-regulated in overexpression of *MeLRR4* in *Arabidopsis* plants than in control plants. *AtICS1*, *AtPDF1.2*, *AtPR1*, and *AtPR5* genes were up-regulated or down-regulated under different plant pathogen infections. These results indicate that overexpression of *MeLRRs* resulted in enhanced resistance simultaneously against pathogenic bacteria and pathogenic fungi, demonstrating the requirement of *MeLRRs* for resistance to plant pathogens.

## Discussion

NBS-LRR proteins play important roles in pathogen recognition and defense response signal transduction (Urbach and Ausubel, 2017). An increasing number of NBS-LRR proteins that conferred resistance to pathogens have been cloned from higher plants (Liu et al., 2017), such as TaRCR1 (Zhu et al., 2017), ZmNBS25 (Xu et al., 2018), GbaNA1 (Li et al., 2018a,b), GhDSC1 (Li et al., 2019), and OsRLR1 (Du et al., 2021). In this study, we found that *MeLRR1*,*-2*,*-3*,*-4* expression could be induced by *Xam* inoculation. Similar expression patterns have been observed in other plant *NBS-LRR* genes, such as *AhRRS5* (Zhang et al., 2017) and *SacMi* (Zhou et al., 2018). *NBS-LRRs* mainly participate in plant resistance against pathogen infection, and we speculated that the up-regulation of *MeLRRs* could help cassava successfully evade *Xam* inoculation.

SA is a secondary messenger for systemic acquired resistance (SAR), and its production in plants represents the successful recognition of pathogen infection and pathogen-associated molecular pattern (PAMP)-triggered immunity (PTI) and effector-triggered immunity (ETI) (Divi et al., 2010; Peng et al., 2021). In cassava, SA also plays an important role in the regulation of cassava resistance to CBB (Liu C. et al., 2018; Chang et al., 2020; Wei et al., 2021a,b) and to whitefly (Irigoyen et al., 2020). Wei et al. (2018) found that *MeHsf3* regulates cassava resistance to cassava bacterial blight through modulation of SA accumulation. Cassava co-chaperones MeHSP90.9 interacts with MeSRS1 and MeWRKY20 to activate SA biosynthesis, accumulation of SA, and thus improve resistance to CBB (Wei et al., 2021b). Therefore, endogenous SA accumulation levels are an indicator of resistance to CBB. We found that the expression levels of *MeLRR* were significantly increased by SA treatment, which showed the similar expression pattern of *NPR1* in *Arabidopsis*, *ZmNBS25* in maize, and *GhDSC1* in cotton. In response to pathogen infection, plant endogenous SA is quickly and strongly induced.

Moreover, multiple transcription activator-like (TAL) effectors and type III effectors (T3Es) of *Xam* regulate plant immune (Castiblanco et al., 2013; Medina et al., 2018). Such as, TALE1_*Xam*_ (Castiblanco et al., 2013), Xop (Arrieta-Ortiz et al., 2013), avrBS2, xopQ, XopR, XopAO1, and similar factors (Bart et al., 2012; Cohn et al., 2016; Medina et al., 2018; Mondal et al., 2020). Flagellin peptide (flg22) treatment regulates the expression of *MebZIP3*, -*5* (Li et al., 2017), *MeBIK1* (Li et al., 2018c), *MeDELLAs* (Li et al., 2018d), *MeWHYs* (Liu W. et al., 2018), and *MeASMT2* (Wei et al., 2017). Moreover, these genes mediated cassava resistance to CBB. Flg22 is a bacterial PAMP. In *Arabidopsis* and tomato, flg22 was used to instead of *P. syringae* and *Xanthomonas* to measure the ROS burst, respectively (de Torres Zabala et al., 2015; Bhattarai et al., 2016). Interestingly, *MeLRRs* regulated ROS burst was induced by flg22 (Zipfel et al., 2004). As a homolog protein of MeLRR3, AtLRRAC1 is induced by flg22 treatment and leads to production ROS and induction of pathogen-responsive genes (Bigeard et al., 2015; Bianchet et al., 2019). Therefore, we hypothesized that *MeLRRs* and effectors of *Xam* conform to the gene for gene theory.

*AtPDF1.2*, *AtPR1*, *AtPR2*, and *AtPR5* are widely known as marker genes for innate immune response (Wang et al., 2017; Xu et al., 2018). *AtICS1* is a key enzyme for SA biosynthesis (Macaulay et al., 2017). *AtTGA3* showed strong affinity for the NPR1 protein (Zhou et al., 2000; Yuan et al., 2009). In pathogenic microorganism infection, the SAR defense response is triggered by elevated SA through an SA-NPR1-TGA-PR1 signaling pathway (Zhang, 2003). Further analysis of gene expression in overexpression of *MeLRR1,−2,−3,−4* at *Arabidopsis* leaves suggested that these genes might exert their function through SA biosynthesis and immune responses. This is similar to the function of *MeHsf3* (Wei et al., 2018), and *MebZIP3, -5* (Li et al., 2017), which were confirmed to regulate cassava resistance against cassava bacterial blight. Hence, we conclude that *MeLRR1,−2,−3,−4* may regulate the plant immune response through SA and ROS accumulation, and the transcription of disease resistance genes. Taken together, the *MeLRR* genes encode a class of NBS-LRR proteins, which controls immunity to *Xanthomonas axonopodis* pv. *manihotis* in cassava. Further investigation of the role of the *MeLRRs* will build an important foundation for future development of resistant cultivars, which may be the most effective means of controlling this devastating disease.

## Materials and Methods

### Plant Materials, Growth Conditions, and Treatments

Cassava (*M. esculenta*), variety South China 124 (SC124), and *N. benthamiana* were cultivated in mixed soil (vermiculite/nutritional soil = 2:1, v.v.) in a greenhouse with 16/8 h light/dark at 28/22°C, 60–70% relative humidity with irradiance of 130–150 uE.m^–2^.s^–1^. *A. thaliana* ecotype Col-0 (Columbia-0) seedlings were cultivated in the mixed soil under fluorescent light (130–150 uE.m^–2^.s^–1^) and were grown under 16/8 h light/dark at 22°C. For axenic growth, *N. benthamiana*, and *A. thaliana* seeds were sterilized (10% NaClO for 1 min, washed five times with sterile water) and sown on half-strength MS (Murashige and Skoog) medium (PhytoTechnology Laboratories, Kansas, United States) with 0.4% agar powder and 2% (w/v) sucrose. The seeds were grown in chambers under 16/8 h light/dark at 22°C and 130–150 uE.m^–2^.s^–1^. For expression analysis, 4-week-old cutting seedlings of cassava leaves were sprayed with 5 mmol/L salicylic acid or *Xam* suspension for 0, 1, 3, 6, 12, or 24 h, and the bacterial solution was diluted to 4 × 10^8^ colony-forming units/mL (cfu/mL) using 10 mmol/L MgCl_2_ with 0.05% Silwet L-77.

### Comprehensive Characterization and Bioinformatics Analysis of *MeLRR* Genes

The sequences of *MeLRR* genes were searched and obtained from the cassava genome database, *M. esculenta* v6.1 (Phytozome v13^1^) (Muñoz-Bodnar et al., 2014; Lozano et al., 2015; Bredeson et al., 2016). The ProtParam tool^2^ was used to predict the number of amino acids, relative molecular mass of protein, isoelectric point, total average hydrophilicity stability index, fat coefficient, and instability index (Gasteiger et al., 2003). Alignments between MeLRRs and other NBS-LRR proteins were performed used DNAMAN 6.0, and the phylogenetic tree was constructed by the neighbor-joining method based on the whole protein sequences and considering 1,000 bootstrap replicates using ClustalW tool and MEGA 7 (Kumar et al., 2016). The 24 NBS-LRR protein amino acid sequences in 13 species were screened based on the principles of encoding nucleotide-binding site (NBS) and leucine-rich repeat (LRR) domains, and were validated through comparisons of the protein basic local alignment search tool (BLASTP) with the National Center for Biotechnology Information (NCBI). The 24 NBS-LRR proteins were derived from *A. thaliana* (CAA0374684.1, CAD5320387.1, CAE6029947.1, NP_181039.1, OAP10808.1, VYS54481.1), *Durio zibethinus* (XP_022746274.1), *H. brasiliensis* (XP_021646775.1, XP_021652057.1, XP_021646749.1, XP_021684995.1, KAF229 5929.1), *J. curcas* (XP_012073222.1, KDP37136. 1. XP_02053 5356.1), *Populus alba* (XP_034892116.1, XP_034896332.1), *P. euphratica* (XP_011001622.1), *P. trichocarpa* (RQO87881.1), *Ricinus communis* (EEF44774.1), *Theobroma cacao* (XP_01796 9995.1), *Vernicia montana* (AMM43068.1), *V. vinifera* (XP_010657.1), and *Ziziphus jujuba* (XP_024924720.1), respectively.

### RNA Extraction, cDNA Synthesis, and Quantitative Real-Time PCR

Total RNA was extracted from three independent pools, and DNA contamination was removed using the Tiangen RNA prep pure plant plus kit (Tiangen Biotech, Beijing, China, Cat# DP441). cDNA synthesis was performed using the Tiangen FastQuant RT kit (Tiangen Biotech, Beijing, China, Cat# KR116) with 20-μl reaction mixture. qRT-PCR analysis was performed using UltraSYBR Mixture (low ROX) (CoWin Biosciences, Beijing, China, Cat# CW0956) in an ABI QuantStudio™ 6 flex Real-Time PCR System (ABI, CA, United States). The PCR cycling conditions were 95°C for 10 min, followed by 40 cycles at 95°C for 15 s and 60°C for 1 min. The *Arabidopsis* and cassava gene transcripts were normalized to the *AtAct2* gene (AT3G18780) and elongation factor 1α (*EF1*α, Me.15G054800) using the comparative 2^–Δ^ ^Δ^ *^Ct^* method, respectively (Livak and Schmittgen, 2001). The qRT-PCR primers of *MeEF1a*, *MePR1* were obtained from Wei et al. (2018), *AtPR1*, *AtPR2*, *AtPR5*, *AtPDF1.2*, *AtICS1*, *AtAct2*, and *BcActA* were obtained from Mhamdi and Noctor (2016), and *AtTGA3* was obtained from Ndamukong et al. (2017), respectively. The qRT-PCR primers of *MeLRRs*, and *AbAct* (JQ671669.1) of *A. brassicicola* were designed by Primer3Plus^3^ to find optimal primers (Untergasser et al., 2007), and then the specificity of the melt curve analyzed performed to determine. In addtion, the qRT-PCR fragments and VIGS fragments are different CDS regions of *MeLRRs*. The primers used are listed in Supplementary Table 2.

### Plasmid Construction and Transient Expression in Plant Leaves

For overexpression, the full-length coding regions of *MeLRR1*,*-2*,*-3*,*-4* were amplified and cloned into the pEGAD vector (Promoter *CaMV35S:GFP*) via appropriate restriction enzyme digestion and T4 DNA ligase. The recombinant plasmids and empty vector were transformed into Agrobacterium GV3101. Then, the *A. tumefaciens* suspension was used to infect the leaves of cassava or tobacco as described by Sparkes et al. (2006) and Zeng et al. (2019). Tobacco leaves injected with Agrobacterium GV3101 for 2 days, the GFP fluorescence and DAPI (4’,6-diamidino-2-phenylindole, Thermo Fisher Scientific, Shanghai, China)-stained cell nuclei were imaged under a fluorescence microscope (Leica TCS SP8, Solms, Germany), with an excitation wavelength of 488 nm and a 505–530-nm bandpass emission filter. Cassava leaves inject with recombinant pEGAD plasmids or empty vector of Agrobacterium GV3101. Then, 3 days later, the cassava leaves were syringe infiltrated with 4 × 10^8^ cfu/mL of pathogenic bacteria *Xam* used for disease resistance assay, include number of *Xam* populations, *MePR1* transcript level, and symptoms of cassava bacterial blight at 0 and 1 dpi, respectively.

VIGS constructs are usually prepared using 300--500 bp partial CDS regions of *MeLRRs* and the online siDirect 2.0^4^ tools (Naito et al., 2009) are available for predicting regions with high siRNA generating capability (Naito et al., 2009; Ui-Tei and Naito, 2013). Zeng et al. (2019) constructs the method about Agrobacterium-mediated Tobacco Rattle Virus (TRV)-based gene silencing in cassava. For VIGS in cassava, the specific CDS fragments of *MeLRR1,−2,−3,−4* were amplified and cloned into the pTRV2 vector through appropriate restriction enzyme digestion and T4 DNA ligase. The recombinant plasmids and empty vectors were transformed into Agrobacterium GV3101. Then, the Agrobacterium suspension, as well as pTRV1, was used to infect the leaves of cassava as previously described (Zeng et al., 2019). At 14 dpi, the new leaves were syringe infiltrated with 4 × 10^8^ cfu/mL of pathogenic bacteria *Xam* used for disease resistance assay. The sequences of primers used for vector construction in this study are listed in Supplementary Table 2.

### *Arabidopsis* Transformation

*Arabidopsis thaliana* ecotype Col-0 was used as wild-type. Overexpressing lines were transformed by floral dip transformation method of *35S:GFP-MeLRR* recombinant plasmids constructs with Agrobacterium GV3101 (Bechtold and Pelletier, 1998). The overexpressing lines were selected by 100 mg/L kanamycin and 20 mg/L glufosinate (Basta; Sangon Biotech. Shanghai, China) resistance and further confirmed by PCR. Single insertion transgenic lines were chosen for further analysis in transgenic third generations (T3).

### Quantification of Endogenous SA Contents

The endogenous SA content in leaves was determined as previously described (Wei et al., 2018). Briefly, leaves were flash-frozen in liquid nitrogen and ground to a very fine powder. SA was extracted from 0.1 g powder using phosphate-buffered solution (PBS, pH 7.4, 0.15 M) on ice. Then, the supernatant was used for SA quantification using a plant SA ELISA (enzyme-linked immunosorbent assay) kit (Jiangsu Meimian Industrial, Jiangsu, China, Cat#HLE01901) according to the manufacturer’s instructions.

### Reactive Oxygen Species Burst Measurements

The ROS burst in leaves was determined as described previously (de Torres Zabala et al., 2015; Chang et al., 2020; Yan et al., 2021). In tomato, flg22 was used to instead of *Xanthomonas* to measure the ROS burst (Bhattarai et al., 2016). Similar methods were applied to study the cassava resistance to *Xam*, such as *MeCAMTA3* (Chang et al., 2020), *MeRAV5* (Yan et al., 2021). Herein, to measure the ROS burst, 48 leaf discs (5 mm in diameter) of cassava were placed in 48 single wells of 96-well black plates and placed in the dark for 12 h in 100 μL double-distilled water. After 12 h, the 48 leaf discs were divided into two groups. In one group, the water was replaced with 100 μL incubation solution containing 0.2 μmol/L luminol (AppliChem, Darmstadt, Germany) and 10 μg/mL horseradish peroxidase (AppliChem, Darmstadt, Germany). In the other group, the water was then replaced with 100 μL incubation solution containing 0.2 μmol/L luminol, 10 μg/mL horseradish peroxidase and 1 μmol/L flg22 (Phyto Technology Laboratories, Lenexa, KS, United States). Luminescence was measured immediately for 30 min using a GloMax 96 Microplate Luminometer (Promega, Madison, WI, United States). Luminescence readout is given in relative light emitting units (RLU).

### Trypan Blue Staining

The cassava or *N. benthamiana* leaves were boiled for 1 min in the trypan blue working solution (100 mL lactic acid, 100 mL glycerol, 100 g phenol, and 0.2 g trypan blue, dissolved in 100 mL distilled water) for 24 h at room temperature (Luo et al., 2017). The leaves were transferred into a chloral hydrate solution (2.5 g/mL) and repeatedly reduced until the background was gone (Luo et al., 2017).

### Pathogen Culture and Disease Assays

The pathogenic bacterium *P. syringae* pv. *tomato* (*Pst*) DC3000 was streaked on LB medium with 50 mg/L of rifampicin at 28°C and shaken to OD_600_ reached 0.6. Thereafter, a fresh bacterial culture of *Pst* DC3000 was diluted to 4 × 10^8^ cfu/mL in 10 mmol/L MgCl_2_ and 0.05% Silwet L-77 and then sprayed on 24-day-old *Arabidopsis* leaves. The *A. brassicicola* and *B. cinerea* strains were cultured on potato dextrose agar (PDA) medium with 2% (w/v) sucrose at 28°C. Conidia were suspended in distilled water for plant infection. Spore suspensions (about 4 × 10^6^ spores/mL) of *A. brassicicola* and *B. cinerea* were sprayed on *Arabidopsis* leaves. The infected plants were grown in an incubator at 90% RH and 22°C. At 0, 2, and 4 dpi, the number of *Pst* DC3000 bacteria was determined, as well as the fungal *actin* gene transcript in leaves of Col-0 and mutants infected with *B. cinerea* and *A. brassicicola* (Veronese et al., 2006; Mhamdi and Noctor, 2016).

### Analysis of Experimental Data

Mean and standard deviations are displayed as representative values for data in the figures. Analysis of variance (ANOVA) with Duncan’s test and Student’s *t-*test were applied to the obtained data with the help of IBM SPSS v20. Statistical significance (*) was set at *p* < 0.05. Each assay contained three independent replicates.

## Data Availability Statement

The original contributions presented in the study are included in the article/Supplementary Material, further inquiries can be directed to the corresponding author.

## Author Contributions

YW and HZ designed the research. HZ did most experimental works and wrote the manuscript. ZY, ZL, and YS did experimental works and database analysis. XL, JW, and GZ did experimental works. YW supervised this project. All authors contributed to the article and approved the submitted version.

## Conflict of Interest

The authors declare that the research was conducted in the absence of any commercial or financial relationships that could be construed as a potential conflict of interest.

## Publisher’s Note

All claims expressed in this article are solely those of the authors and do not necessarily represent those of their affiliated organizations, or those of the publisher, the editors and the reviewers. Any product that may be evaluated in this article, or claim that may be made by its manufacturer, is not guaranteed or endorsed by the publisher.

## Footnotes

1
https://phytozome-next.jgi.doe.gov/

2
http://web.Expasy.org/protpasam/

3
https://www.bioinformatics.nl/cgi-bin/primer3plus/primer3plus.cgi

4
http://siDirect2.RNAi.jp/
