# Supplementary material for: The Cassava NBS-LRR Genes Confer Resistance to Cassava Bacterial Blight
Source: Front Plant Sci. 2022 Feb 1;13:790140. doi: 10.3389/fpls.2022.790140 (PMC8844379; doi:10.3389/fpls.2022.790140)

**SUPPLEMENTARY MATERIAL**

**ARITICLE TITLE: The cassava NBS-LRR genes confer resistance to cassava bacterial blight**

**AUTHORS:** He Zhang | Zi Ye | Zhixin Liu | Yu Sun | Xinyu Li | Jiao Wu | Guangzhen Zhou | Yinglang Wan^*^

Additional supporting information may be found online in the Supplenmentary Material section.

**Supplementary Table 1** The detailed information of *MeLRRs* genes in cassava

| **Gene** | **Sequence ID**  **（Manes.）** | **Number of amino acids** | **MW/KDa** | **pI** | **GRAVY** | **Aliphatic index** | **Instability index** |
| --- | --- | --- | --- | --- | --- | --- | --- |
| *MeLRR1* | 11G053000.1 | 1020 | 116.47 | 6.02 | -0.2 | 98.29 | 43.20 |
| *MeLRR2* | 03G071700.1 | 927 | 102.02 | 5.93 | -0.032 | 102.61 | 32.82 |
| *MeLRR3* | 13G036800.1 | 1130 | 128.11 | 6.11 | -0.111 | 101.46 | 45.81 |
| *MeLRR4* | 07G107800.1 | 1180 | 134.23 | 6.07 | -0.157 | 101.53 | 48.96 |

**Supplementary Table 2 Primers used in this study**

| **Primers name** | **Primers sequence (5'-3')** | **Application** |
| --- | --- | --- |
| MeLRR1F | ATGGGTAATTGCTTCTCAATTCAA | MeLRR1 amplification |
| MeLRR1R | CTAGATATCAAAAAAGTATGGGGGC |
| MeLRR2F | ATGGATACCAGTAGTCGCTTAAATTC | MeLRR2 amplification |
| MeLRR2R | CTACAAGATTCTGTCACCTCCAGGG |
| MeLRR3F | ATGGCGGACGCATTGGTA | MeLRR3 amplification |
| MeLRR3R | TCAATTGACATTCTGGAAATTGATC |
| MeLRR4F | ATGGCTCTGTCGGTTGTGGG | MeLRR4 amplification |
| MeLRR4R | TCAATTGATCTTGTGGAAATTAACTTT |
| MeLRR1GE-F | GCTGCGGCAGCGGCCGAATTCATGGGTAATTGCTTCTCAATTCAA | MeLRR1 for pEGAD |
| MeLRR1GB-R | TTATCTAGATCCGGTGGATCCCTAGATATCAAAAAAGTATGGGGGC |
| MeLRR2GE-F | GCTGCGGCAGCGGCCGAATTCATGGATACCAGTAGTCGCTTAAATTC | MeLRR2 for pEGAD |
| MeLRR2GB-R | TTATCTAGATCCGGTGGATCCCTACAAGATTCTGTCACCTCCAGGG |
| MeLRR3GE-F | GCTGCGGCAGCGGCCGAATTCATGGCGGACGCATTGGTA | MeLRR3 for pEGAD |
| MeLRR3GB-R | TTATCTAGATCCGGTGGATCCTCAATTGACATTCTGGAAATTGATC |
| MeLRR4GE-F | GCTGCGGCAGCGGCCGAATTCATGGCTCTGTCGGTTGTGGG | MeLRR4 for pEGAD |
| MeLRR4GB-R | TTATCTAGATCCGGTGGATCCTCAATTGATCTTGTGGAAATTAACTTT |
| 35S | GACGCACAATCCCACTATCC | pEGAD |
| NOS | GATAATCATCGCAAGACCGG | pEGAD |
| MeLRR1VE-F | CCGGAATTCAAGAAAAAAGTTGTCATAGC | MeLRR1 for pTRV2 |
| MeLRR1VK-R | CGGGGTACCCAAGCCCACTGTTGGCTCAGC |
| MeLRR2VE-F | CCGGAATTCCTTCTCATTTTTTATTTCTC | MeLRR2 for pTRV2 |
| MeLRR2VK-R | CGGGGTACCAAATGACGAGAAAGAGAGGT |
| MeLRR3VE-F | CCGGAATTCACCTTGATATCTGTCAATGG | MeLRR3 for pTRV2 |
| MeLRR3VK-R | CGGGGTACCTGCTGCTATTTTCATCATTTC |
| MeLRR4VE-F | CCGGAATTCAAGATGTTGGCTATTAACGG | MeLRR4 for pTRV2 |
| MeLRR4VK-R | CGGGGTACCATCAGATAACAGCAAATTTA |
| qMeLRR1F | GTAGAGAGAGTGATCCGTGATG | MeLRR1 for qRT-PCR |
| qMeLRR1R | CAGCCGATCAACTTCATTTAGG |
| qMeLRR2F | TGCAGCTGTCAATGGAATTAAC | MeLRR2 for qRT-PCR |
| qMeLRR2R | GCTGATTTCTTGACAGGTTCAG |
| qMeLRR3F | CAAGTTAAAAACGCTTCGCATC | MeLRR3 for qRT-PCR |
| qMeLRR3R | CAAATTAGGACACTCCCTGACT |
| qMeLRR4F | GCTTTGTATGAAGCCGATGATT | MeLRR1 for qRT-PCR |
| qMeLRR4R | CATCTGATCTCCAAAGGTACGA |
| qMeEF1aF | TGAACCACCCTGGTCAGATTGGAA | MeEF1a for qRT-PCR |
| qMeEF1aR | AACTTGGGCTCCTTCTCAAGCTCT |
| qMePR1F | TCGTCCATTGCCTAAGAT | MePR1 for qRT-PCR |
| qMePR1R | GTCACCAGCTCGTTGATT |
| qAtPR1-F | AGGCTAACTACAACTACGCTGCG | AtPR1 for qRT-PCR |
| qAtPR1-R | GCTTCTCGTTCACATAATTCCCAC |
| qAtPR2-F | TCAAGGAGCTTAGCCTCACC | AtPR2 for qRT-PCR |
| qAtPR2-R | CGCCTAGCATCCCGTAGC |
| qAtPR5-F | GCCCTACCACCGTCTGG | AtPR5 for qRT-PCR |
| qAtPR5-R | CGGGAAGCACCTGGAGTC |
| qAtPDF1.2-F | CCAAACATGGATCATGCAAC | AtPDF1.2 for qRT-PCR |
| qAtPDF1.2-R | CACACGATTTAGCACCAAAGA |
| qAtICS1-F | TTGGTGGCGAGGAGAGTG | AtICS1 for qRT-PCR |
| qAtICS1-R | CTTCCAGCTACTATCCCTGTCC |
| qAtTGA3-F | GAACCACTACGCAAATCTCTTC | AtTGA3 for qRT-PCR |
| qAtTGA3-R | AAGAAGCGTTCAGTTGAAGTTC |
| qAtAct2F | AAGCTCTCCTTTGTTGCTGTT | AtAct2 for qRT-PCR |
| qAtAct2R | GACTTCTGGGCATCTGAATCT |
| qBcActA-F | ACTCATATGTTGGAGATGAAGCGCA | BcActA for qRT-PCR |
| qBcActA-R | AATGTTACCATACAAATCCTTACGGA |
| qAbAct-F | CACGGTGTCGTTACCAACTG | AbAct for qRT-PCR |
| qAbAct-R | AGCGTTGAAGGTCTCGAAAA |

**Supplementary Figure 1** Phylogenetic tree of cassava MeLRRs. The phylogenetic tree was constructed by the neighbor-joining method based on whole protein sequences and considering 1,000 bootstrap replicates using the ClustalW tool and MEGA 7.


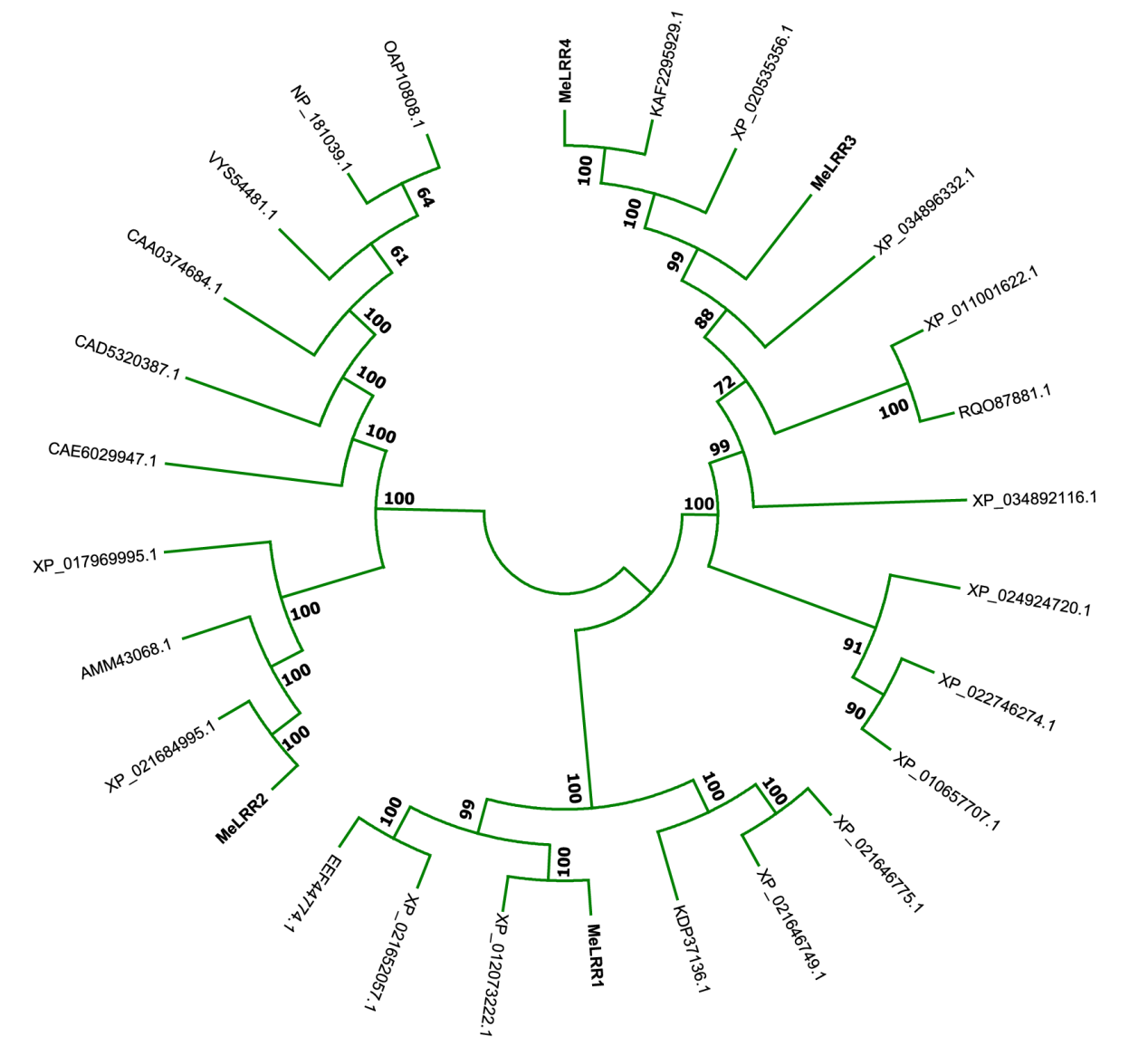


**Supplementary Figure 2** The relative transcript levels of *MeLRRs* in *MeLRR*-silenced leaves. After silenced the target gene 14 days, the new cassava leaves were used for analysis. The relative transcript level of *MeLRRs* in the pTRV control leaves was normalized to 1.0. Asterisks (*) indicate significant differences at *p*<0.05. dpi is days post-infection.


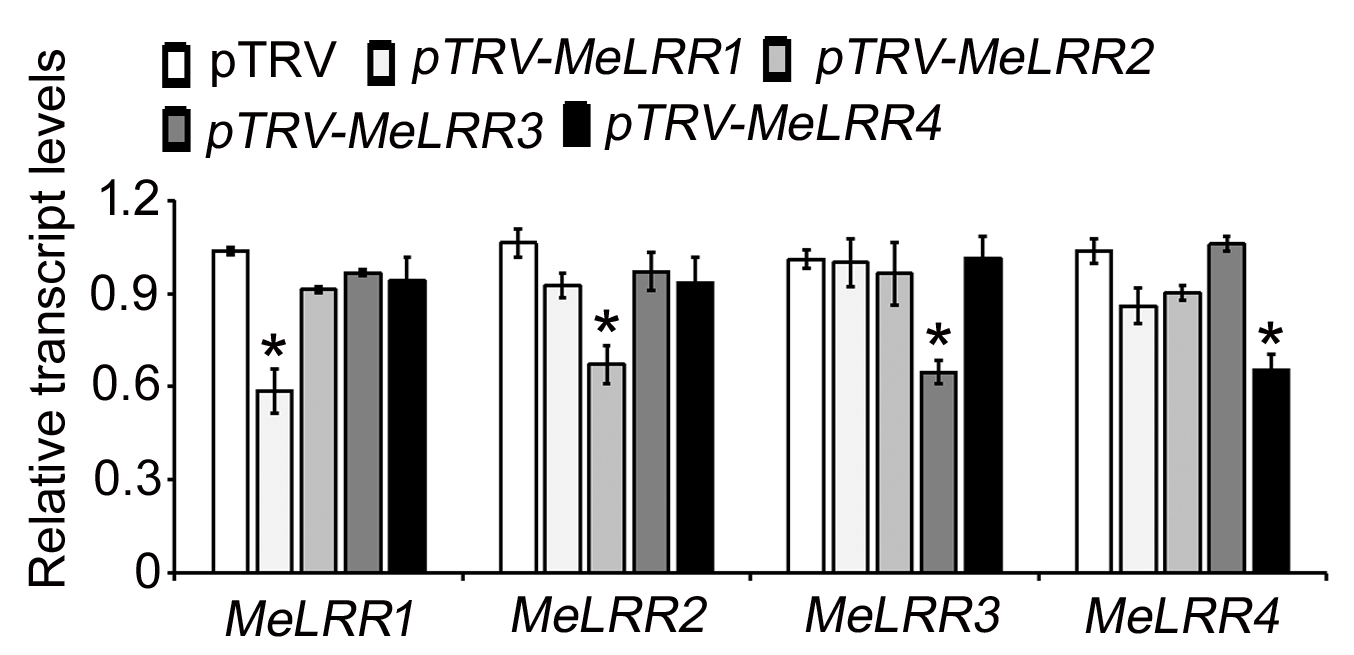


**Supplementary Figure 3** The transcript level of *MeLRRs* and *MePR1* in *MeLRR-1,-2,-3,-4*-silenced plants. The cassava leaves simultaneous silencing four target genes (*MeLRR-1*,*-2*,*-3*,*-4*) by VIGS with Agrobacterium GV3101. After silenced the target gene 14 days, the new cassava leaves were used for analysis. Asterisks (*) indicate significant differences at *p*<0.05.


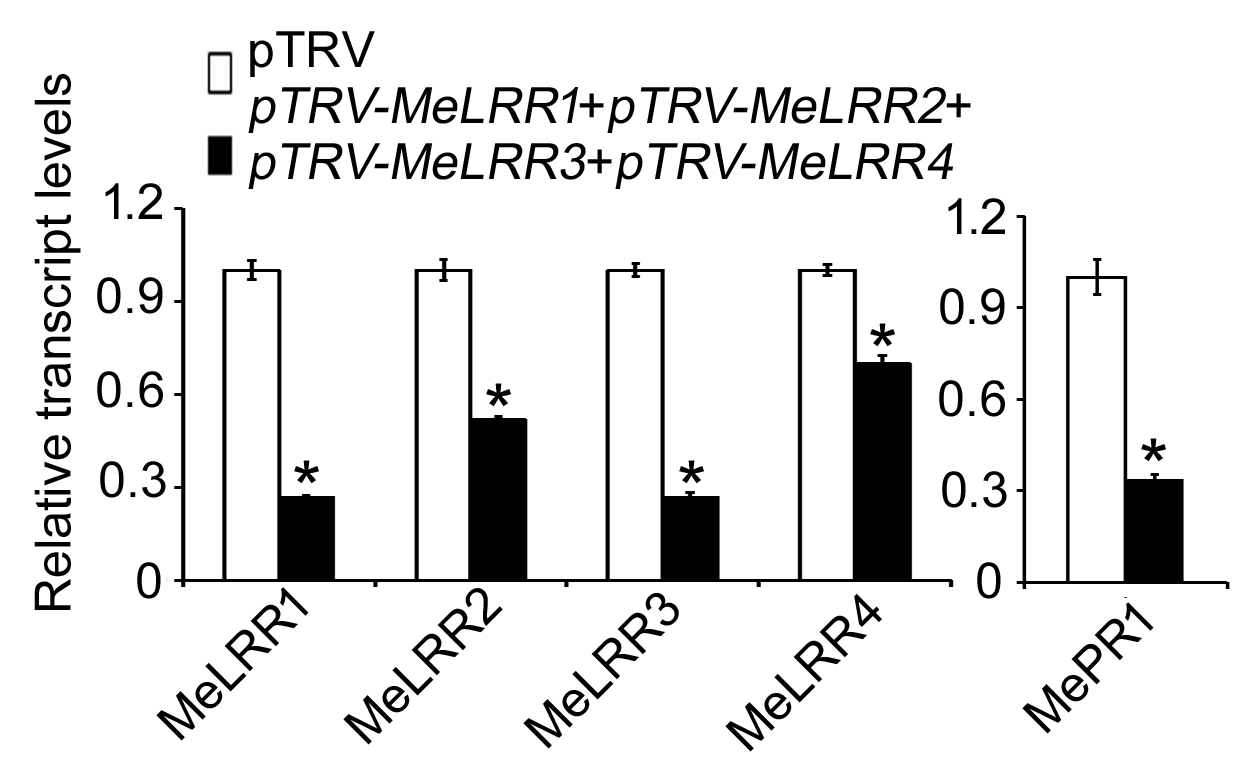


**Supplementary Figure 4** The transcript level of *MeLRRs* in overexpression plants. After over-expressed 3 days, the infected cassava leaves were used for transcript level analysis. The relative transcript level of *MeLRRs* in the *35S::GFP* control leaves was normalized to 1.0. Asterisks (*) indicate significant differences at *p*<0.05. dpi is days post-infection.


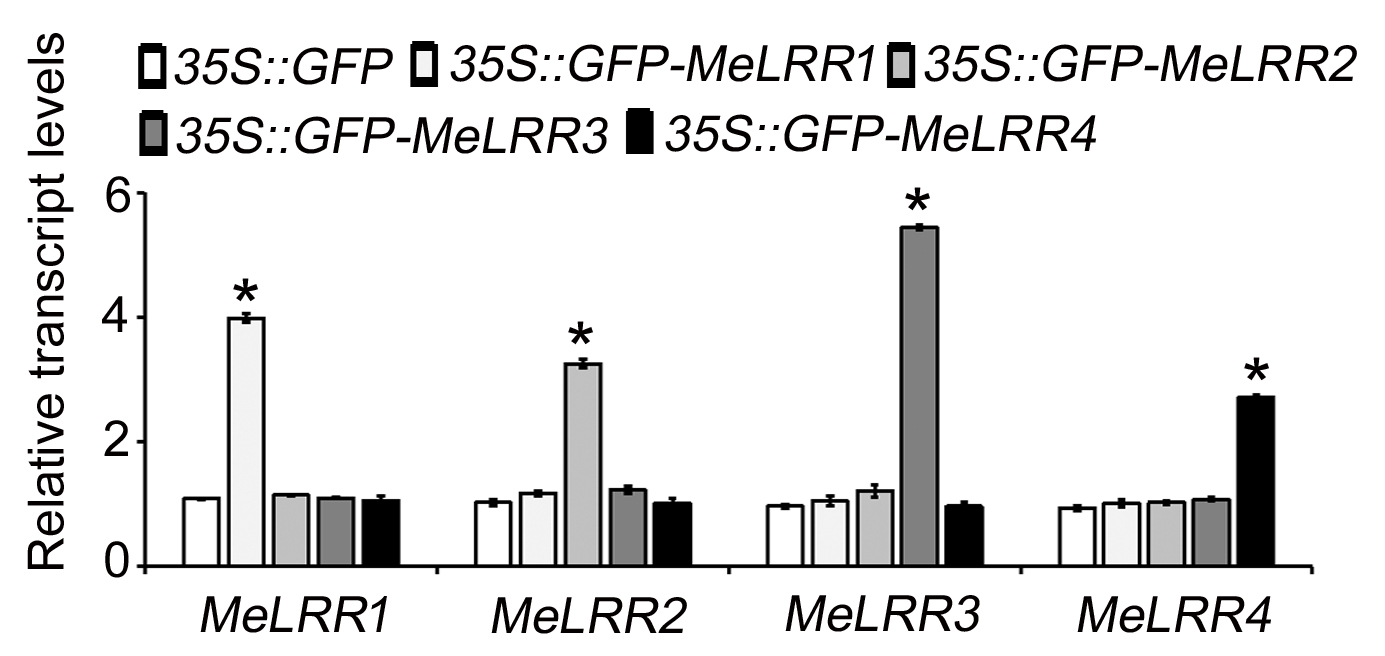


**Supplementary Figure 5** The transcript level of *MeLRRs* and *MePR1* in co-overexpression *MeLRR-1,-2,-3,-4* lines. The cassava leaves transiently expressed *35S::GFP*, or simultaneous transiently expressed *35S::GFP-MeLRR1,-2,-3,-4* with Agrobacterium GV3101. After over-expressed 3 days, the infected cassava leaves were used for transcript level analysis. Asterisks (*) indicate significant differences at *p*<0.05.


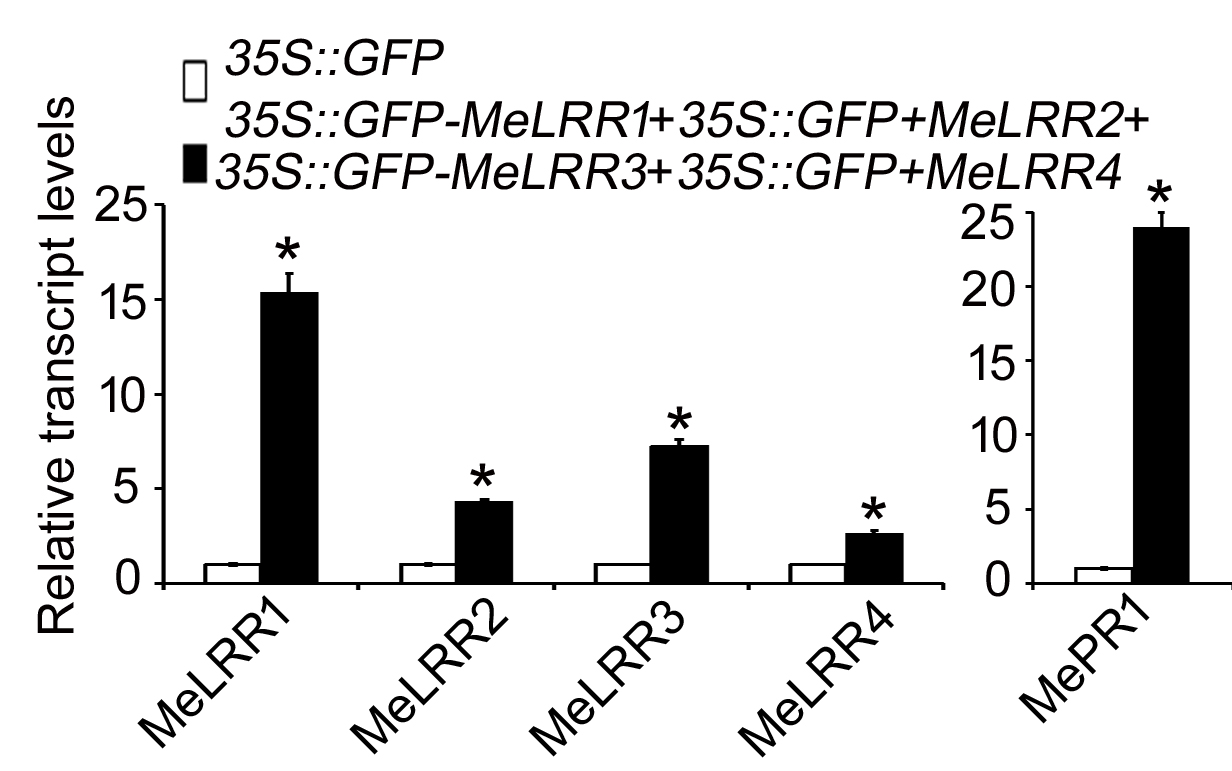


**Supplementary Figure 6** The cell death phenotype of cassava and tabacco (*N. benthamiana*) leaves over-expression *MeLRRs.*. The cassava and *N. benthamiana* leaves transiently expressed *35S::GFP*, *35S::GFP-MeLRR1*,*-2*,*-3*,*-4* with Agrobacterium GV3101. The leaves were photographed at 0 dpi, 2 dpi and were stained with Trypan Blue. dpi is days post-infection.


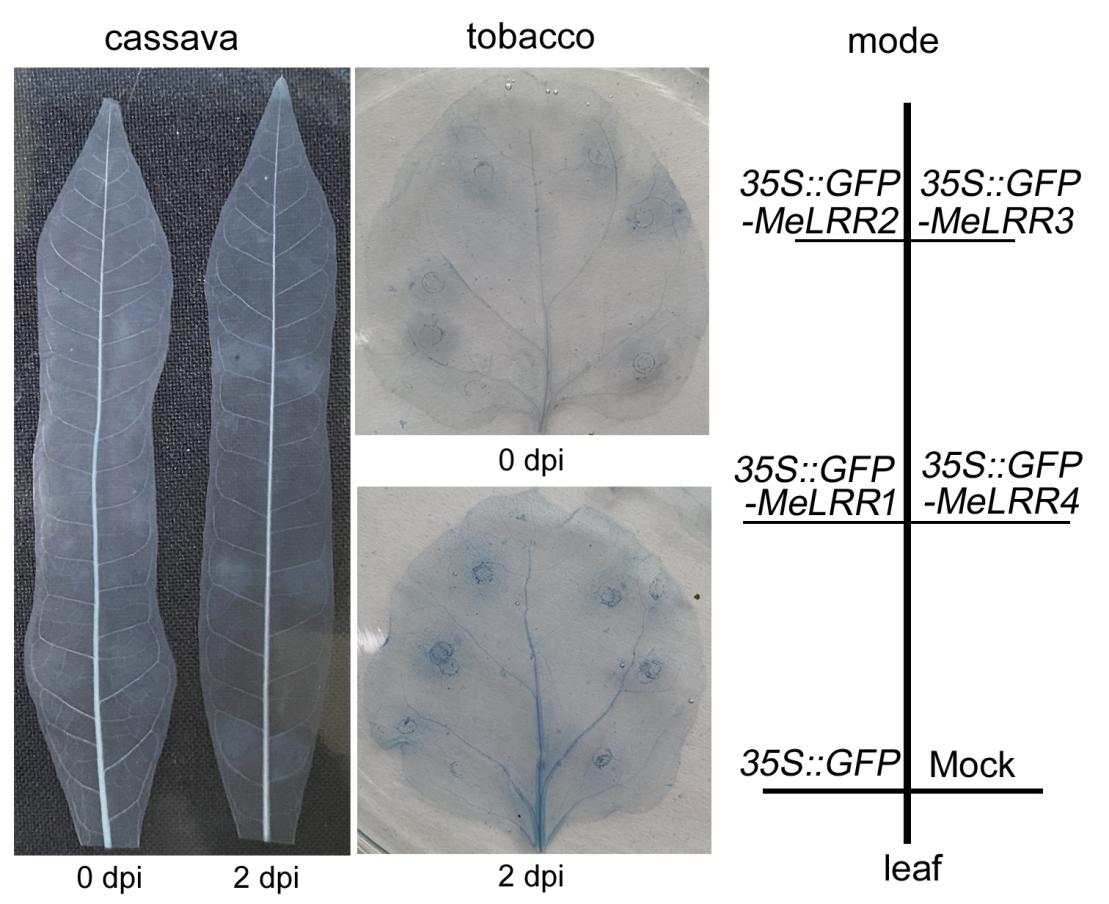


**Supplementary Figure 7** Overexpression of *MeLRRs* in *Arabidopsis* increases the SA content in leaves based on ELISA. Overexpression *Arabidopsis* seedings were cultivated in the mixed soil under fluorescent light (130–150 uE.m^-2^.s^-1^) and were grown under 16/8 h light/dark at 22°C for 24 days. Asterisks (*) indicate significant differences at *p*<0.05. Col-0 is *A. thaliana* ecotype Columbia-0. #1 and #2, #3 and #4, #5 and #6, #7 and #8 are overexpression of MeLRR3 in *A. thaliana* Col-0 lines, respectively.


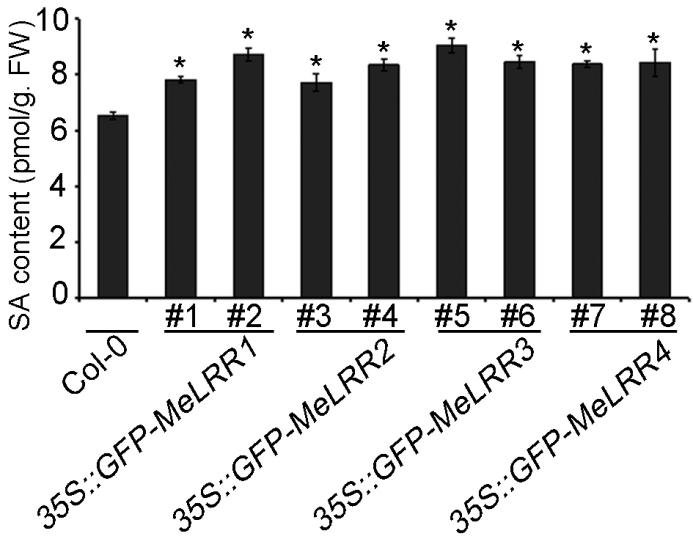


**Supplementary Figure 8** *MeLRR* genes in overexpressing *Arabidopsis* regulate the transcriptional of disease-related genes. The transcript levels of disease-related genes in overexpressing *Arabidopsis* leaves infected by *A. brassicicola*, *B. cinerea*, and *P. syringae* pv. *tomato* for 2 dpi and 4 dpi. The transcript levels of all genes were the control of the overexpressing *Arabidopsis* leaves that were not infected by pathogens, and the control transcript levels were set to 1. Asterisks (*) indicate significant differences at *p*<0.05. dpi is days post-infection.


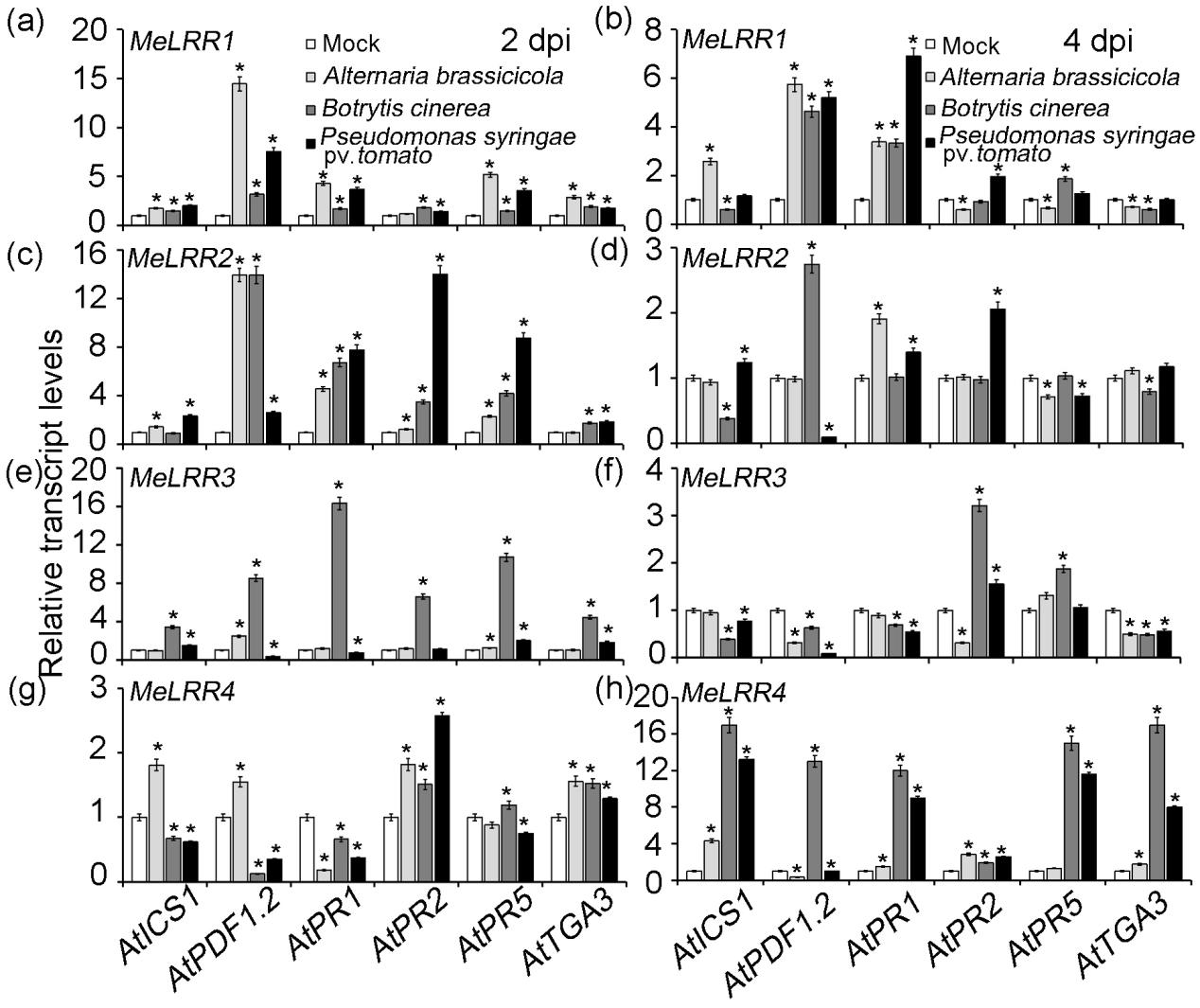

Supplement: Supplementary file 1 [file Data_Sheet_1.doc]
